# Special Issue “Atherosclerosis: From Molecular Biology to Therapeutic Perspective: 6th Edition”

**DOI:** 10.3390/ijms27135806

**Published:** 2026-06-26

**Authors:** Ida Daniela Perrotta

**Affiliations:** Department of Biology, Ecology and Earth Sciences, Centre for Microscopy and Microanalysis (CM2), University of Calabria, Arcavacata di Rende, 87036 Cosenza, Italy; ida.perrotta@unical.it or idaperrotta@yahoo.it

Atherosclerosis remains a leading cause of morbidity and mortality worldwide, representing a complex and multifactorial disease at the intersection of aging, metabolism, inflammation, and environmental exposures. The contributions to this Special Issue highlight a major conceptual transition in the field from a disease-centric perspective to an integrated, systems-level understanding that encompasses molecular mechanisms, biomarker discovery, and preventive strategies.

A key theme emerging from these studies is the heterogeneity of aging trajectories. Cognitive decline, often accompanying vascular aging, is influenced by genetic variability, yet the role of canonical polymorphisms remains nuanced. Evidence from longitudinal analyses challenges the traditionally protective view of certain alleles, such as APOE ε2, emphasizing sex-specific and context-dependent effects. This aligns with previous studies demonstrating the complex influence of APOE variants on neurodegeneration and aging [1,2,3]. Similarly, inconsistent associations reported for BDNF polymorphisms further support the need for integrative approaches to genetic contributions in cognitive aging [1].

Vascular aging is increasingly recognized as a central driver of both cardiovascular and neurodegenerative diseases. Structural and functional changes, including endothelial dysfunction, arterial stiffness, and vascular remodeling, contribute to disease susceptibility even in the absence of traditional risk factors [4,5]. These alterations are closely linked to cognitive impairment and dementia, reinforcing the concept of a vascular continuum underlying brain aging [6,7].

The identification of molecular biomarkers represents a cornerstone of current research efforts. Multi-omics approaches, integrating transcriptomic and epigenomic data, are expanding our understanding of coronary artery disease (CAD) and enabling the identification of regulatory networks involved in disease progression [8,9]. In parallel, extracellular vesicle-derived microRNAs have emerged as promising non-invasive biomarkers reflecting key pathophysiological processes, including inflammation and lipid metabolism [10,11]. These findings underscore the growing relevance of precision medicine in cardiovascular research.

In addition to nucleic acid-based biomarkers, circulating proteins and inflammatory mediators play a crucial role in disease pathogenesis. Adropin has been implicated in endothelial function and metabolic regulation, with reduced levels associated with increased cardiovascular risk [12,13]. Likewise, chemokines such as MCP-1 contribute to monocyte recruitment and plaque instability, linking metabolic dysfunction and inflammation to atherosclerosis progression [14,15].

At the mechanistic level, oxidative stress, macrophage activation, and foam cell formation remain central to atherogenesis [16]. Recent evidence highlights the importance of metabolic regulators such as AMP-activated protein kinase (AMPK), which modulates inflammation and lipid metabolism [17]. Furthermore, the endothelin system plays a significant role in vascular homeostasis, with dysregulation contributing to hypertension and atherosclerotic progression [18].

Beyond molecular mechanisms, a broader systems perspective is essential. The exposome framework emphasizes the cumulative impact of environmental exposures on disease risk [19], while the concept of syndemics highlights the interaction between biological, environmental, and social determinants of health [20]. These perspectives are particularly relevant given the growing evidence linking cardiovascular disease with other chronic conditions, including cancer [21].

A critical implication of these advances is the opportunity for early detection and intervention. Non-invasive imaging techniques, such as coronary artery calcium scoring and carotid intima–media thickness, combined with circulating biomarkers, enable the identification of subclinical disease stages [22,23]. This supports a shift from reactive treatment toward proactive prevention.

In this context, the concept of a “preventome” emerges as a unifying framework, integrating molecular, clinical, and environmental data to enable early risk stratification and targeted intervention [24]. Such an approach is essential to reduce the burden of atherosclerosis and promote healthy aging.

In conclusion, this Special Issue provides a comprehensive overview of the molecular and systemic mechanisms underlying atherosclerosis, highlighting novel biomarkers and therapeutic targets. By integrating insights across disciplines, these contributions support the transition toward precision and preventive cardiovascular medicine.
